# A Multidimensional Particle Swarm Optimization-Based Algorithm for Brain MRI Tumor Segmentation

**DOI:** 10.3390/s25092800

**Published:** 2025-04-29

**Authors:** Zsombor Boga, Csanád Sándor, Péter Kovács

**Affiliations:** 1 Faculty of Mathematics and Computer Science, Babeș-Bolyai University, 400084 Cluj-Napoca, Romania; csanad.sandor@ubbcluj.ro; 2 Faculty of Informatics, Eötvös Loránd University, 1117 Budapest, Hungary; kovika@inf.elte.hu

**Keywords:** multidimensional particle swarm optimization, clustering, image segmentation, adaptive number of segments, brain tumor segmentation, magnetic resonance imaging, random forest classifier

## Abstract

Particle Swarm Optimization (PSO) has been extensively applied to optimization tasks in various domains, including image segmentation. In this work, we present a clustering-based segmentation algorithm that employs a multidimensional variant of PSO. Unlike conventional methods that require a predefined number of segments, our approach automatically selects an optimal segmentation granularity based on specified similarity criteria. This strategy effectively isolates brain tumors by incorporating both grayscale intensity and spatial information across multiple MRI modalities, allowing the method to be reliably tuned using a limited amount of training data. We further demonstrate how integrating these initial segmentations with a random forest classifier (RFC) enhances segmentation precision. Using MRI data from the RSNA-ASNR-MICCAI brain tumor segmentation (BraTS) challenge, our method achieves robust results with reduced reliance on extensive labeled datasets, offering a more efficient path toward accurate, clinically relevant tumor segmentation.

## 1. Introduction

Magnetic resonance imaging (MRI) is widely recognized as a powerful and frequently employed medical imaging modality, especially for detailed examinations of the brain and other soft tissues. Unlike computed tomography (CT), which utilizes ionizing radiation, MRI operates by placing the patient’s body within a strong magnetic field and subjecting it to radiofrequency pulses, inducing resonance in hydrogen protons. These protons emit electromagnetic signals that can be recorded and processed, resulting in high-contrast images without the risks associated with ionizing radiation [1,2].

During a single MRI session, multiple scans are typically acquired under different protocols, creating what can be viewed as various “channels” of the same subject. For instance, T1-weighted images highlight fat-rich tissues and acute hemorrhages, while contrast-enhanced T1 (T1CE) images emphasize tumors or inflammatory processes by administering an intravenous contrast agent. T2-weighted images excel at visualizing fluid-rich or edematous regions, and FLAIR (Fluid Attenuated Inversion Recovery) suppresses signal contributions from cerebrospinal fluid, thereby accentuating pathological changes such as edema [1,2,3].

Image segmentation constitutes a pivotal task within computer vision, involving the separation of structurally distinct parts of an image. This process encompasses both unsupervised and supervised approaches. The choice of method is influenced by various factors, such as the color depth of the image (whether it is in color or grayscale), the desired segmentation accuracy, and the availability of pre-segmented ground truth (GT) data.

Despite significant potential to save both time and clinical resources, most healthcare institutions have not yet fully adopted automated or semi-automated segmentation approaches. In one study [4], a partially automated segmentation system was shown to reduce the total workflow time from an average of 479 s (manual) to 167 s while maintaining comparable accuracy and requiring less physical effort. Moreover, automated segmentation can mitigate variability between different observers as well as within the same observer over time, improving diagnostic precision [5]. This reliability is particularly important for conditions such as gliomas, where early and accurate identification of tumor growth patterns can enhance intervention strategies and potentially improve patient survival rates [6]. It is important to note that target volume definition is the largest source of uncertainty in modern radiotherapy, with both imaging-related and patient-related factors contributing significantly to inter- and intra-observer variations [7]. Therefore, while marginal improvements may suffice for tumor detection, higher segmentation accuracy becomes critical for surgical or radiotherapy planning, where even small errors can adversely affect treatment outcomes.

The precise segmentation of brain tumors has been the focus of intense research [3], mainly due to the challenges involved in achieving reliable results. A primary challenge lies in the diversity of images produced by various MRI devices [8]. Moreover, brain tumors present challenges arising from noise, tissue variability, and indistinct boundaries between tumor and non-tumor tissues. This complexity is compounded by variations in signal intensity across different imaging sequences (e.g., FLAIR, T1, T1C, and T2) capturing the same brain anatomy [3]. Furthermore, the corresponding information may not always be located in the exact same position but, instead, in close proximity [9].

These challenges suggest that deep learning (DL) algorithms may provide ideal solutions. However, this is an overly narrow perspective, as these models require a significant amount of annotated data from medical experts, which is time-consuming and costly to produce. This demand for data, coupled with the lack of interpretability, limits the practical applicability of DL algorithms. Moreover, well-performing DL systems often demand substantial computational capacity, which may not be available in all institutions. For example, the 2021 RSNA-ASNR-MICCAI brain tumor segmentation challenge winner (BraTS2021), achieving a mean Dice score of 92.77%, used a modified U-Net model [10] with 15.729 million trainable parameters. Training this model required 1251 segmented voxel images of the brain.

Recent advances in medical image segmentation harness foundation models and frameworks such as nnU-Net [11] to achieve robust performance. Transfer learning empowers these models to leverage high-level features learned from large, annotated datasets—such as adult glioma cohorts—thereby enabling rapid adaptation to new, smaller target datasets with minimal tuning [11]. This approach enhances generalizability and reproducibility across multi-institutional data. However, it remains computationally intensive, requiring substantial GPU resources for both pretraining and fine-tuning. Moreover, while transfer learning is designed to tailor pre-trained models to new domains, it still necessitates careful calibration to address domain-specific nuances; additionally, the “blackbox” nature of deep models limits interpretability, complicating clinical integration and heightening the risk of overfitting when fine-tuning is performed on small datasets.

Other studies have shown [9] that the size and diversity of training and testing datasets significantly impact performance metrics. For example, an RFC-based approach trained on 5/6 of the available 335 images achieved a mean Dice score of 85.16% for high-grade gliomas (HGG) and 84.79% for low-grade gliomas (LGG) with the best model [9].

Traditional image processing methods are valuable in scenarios where labeled data are scarce and also contribute significantly to enhancing performance and reducing memory and computational costs in other cases. For example, in [12], a supervised segmentation method based on DL and attention mechanisms is presented. This method requires defining a region of interest (ROI), which the study accomplishes through histogram Z-normalization and a combination of thresholding techniques applied to various types of MRI images. This method reached a mean Dice score of 92.03%.

Focusing on unsupervised methods, there exists a plethora of image segmentation algorithms, including threshold-based approaches [13,14] (e.g., Otsu’s method, K-means clustering algorithm) or metaheuristic-based threshold algorithms, as presented in [15,16]. In [17], multi-swarm Particle Swarm Optimization (PSO) was employed to fit a sum of Gaussian probability density functions to brain MRI histograms to determine optimal multimodal thresholds. In general, these methods are unsuitable for object detection because they rely solely on pixel intensity, often resulting in scattered pixels across the image that do not correspond to a single object. This contradicts our objective of accurately identifying and delineating individual objects within the image. Another disadvantage is that these methods are typically designed to determine a fixed number of thresholds, which may not be easily adaptable to different images. This can lead to insufficient separation of objects or overly fine segmentation, resulting in objects being inadequately separated or divided into multiple parts.

## 2. Key Contributions

This work introduces a novel hybrid segmentation framework that effectively combines unsupervised and supervised learning, offering reliable performance with limited labeled data. Our main contributions are as follows:

- We propose a multidimensional particle swarm optimization (MDPSO)-based clustering method that dynamically adapts the number of clusters, ensuring generalizability across heterogeneous and non-synthetic MRI data;
- A fitness function has been developed, integrating both pixel intensity and Euclidean distance measures to enhance segmentation accuracy;
- Incorporating features derived from MDPSO-based unsupervised clustering into a supervised learning framework using an RF classifier yields improved performance compared to traditional supervised approaches under equivalent training conditions;
- The proposed methodology bridges the gap between fully supervised and unsupervised methods, offering a more interpretable and resource-efficient alternative for medical image segmentation.

## 3. Methodology

**Figure 1:**
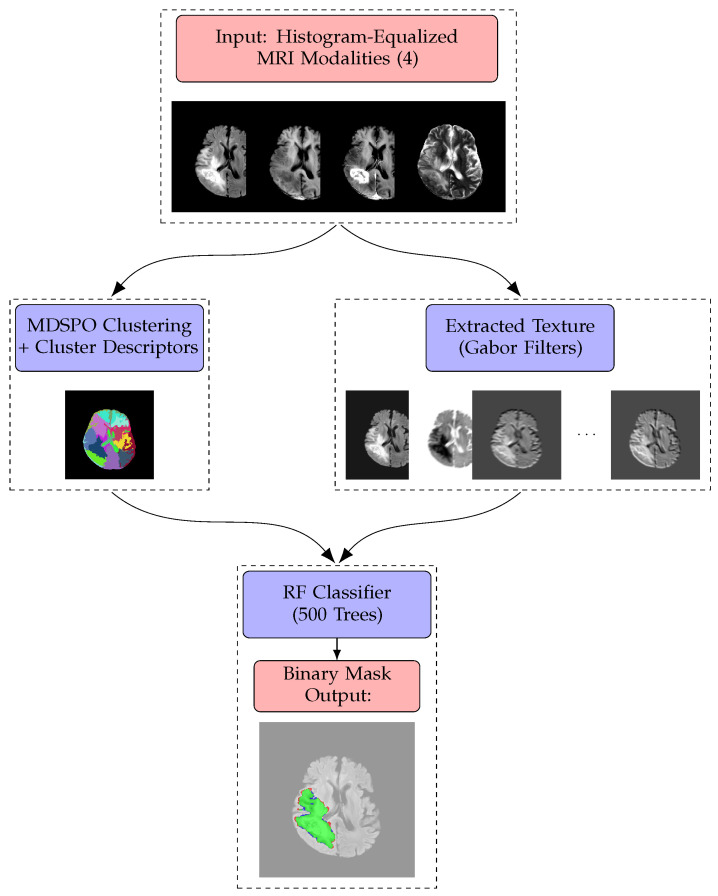
Workflow of the proposed method: Histogram-equalized MRI modalities are input; features are extracted during both training and evaluation using MDPSO clustering and GFs), combined in an RFC for learning, and applied to segmentation prediction and evaluation against the GT.

In this paper, we propose a segmentation methodology (see, e.g., Figure 1) that is capable of adapting to different image domains without requiring a large set of pre-segmented GT images. The unsupervised part of the method dynamically determines the optimal number of clusters for a given image, which is particularly beneficial for object segmentation tasks, such as tumor delineation in multispectral MRI data. Using an approach inspired by MDPSO and ideas drawn from K-means [18] and mean shift-based methods [19], the algorithm can robustly navigate a high-dimensional parameter space encompassing both spatial coordinates and multiple intensity modalities.

The proposed segmentation method, detailed in Section 3.2, interprets an input MRI image as a feature space where each pixel is represented by a vector combining spatial coordinates and intensity values. Utilizing the MDPSO Algorithm 1, pixels are grouped into clusters that capture contextual information based on intensity patterns and spatial arrangements. The fitness function ρ(.,.) Algorithm 2 evaluates these cluster configurations by assessing the distribution of pixels and their cumulative errors relative to cluster centroids, thereby promoting balanced segmentation and avoiding trivial outcomes with inappropriate numbers of clusters.

From these clusters, a comprehensive feature set is derived, encompassing intensity, geometric, contextual, and fractal properties. Intensity features—including mean, standard deviation, contrast, and homogeneity—characterize the statistical distribution within each cluster. Geometric features such as cluster size, bounding box dimensions, aspect ratio, density, perimeter-to-area ratio, and eccentricity describe the shape and spatial structure of the clusters. Additionally, the fractal dimension, calculated using the box-counting method, quantifies boundary complexity, enhancing the representation of cluster-level characteristics. To further refine the feature representation, Gabor filters (GFs) are applied following the initial unsupervised clustering. These filters extract local texture patterns, capturing fine-scale structures and edges that intensity-based features may overlook. The integration of Gabor-derived texture features with region-based descriptors from clustering results in a robust and enriched feature set that encapsulates both pixel-level and region-level properties.
**Algorithm 1:** MDPSO Algorithm
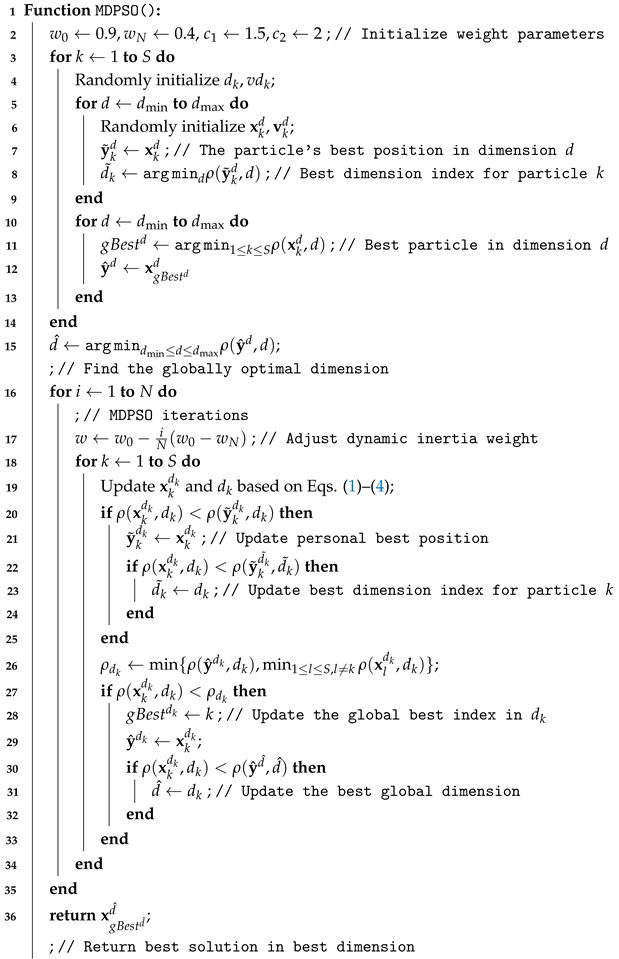


This enhanced feature set is then utilized by a random forest classifier (RFC) to achieve precise segmentation. As demonstrated in [9], RFCs are effective at modeling complex non-linear relationships between features. The combination of local texture features and region-based attributes facilitates improved class separability. Moreover, the ensemble nature of the RFC mitigates overfitting, handles heterogeneous feature distributions, and enhances generalization capabilities, resulting in a stable and flexible segmentation solution.

In general, the method integrates unsupervised clustering with contextual feature extraction and supervised classification to achieve accurate MRI image segmentation. By embedding both local and global contextual information through dynamic cluster-based representations and texture analysis, the approach overcomes the limitations of per-pixel classification methods, as noted in [20]. This comprehensive methodology ensures that the final segmentation adapts to the complexity of the image content, yielding results that are both detailed and contextually coherent.
**Algorithm 2:** Fitness Function Computation for 2D/3D Images
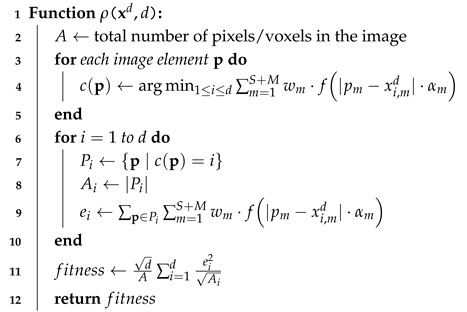


### 3.1. The PSO and MDPSO Algorithms

Swarm intelligence entails collective decision-making, communication, self-organization, and division of labor, surpassing the capabilities of individual agents. This collective behavior ensures the efficient achievement of global goals through distributed tasks and diverse roles among swarm members. The basic PSO algorithms [21] emulate the behavior of natural swarms to solve optimization problems. They offer versatility across various optimization tasks but may not always guarantee the best solution due to their metaheuristic nature. PSO algorithms efficiently explore large search spaces and yield results quickly, making them advantageous over deterministic methods. The multidimensional particle swarm optimization (MDPSO) [22] algorithm inherits the beneficial properties of the basic PSO but significantly extends its capabilities. The algorithm, following the PSO logic, automatically determines the optimal dimension of the solution space without requiring excessive iterations across all possible dimensions. These algorithms are widely applied in various fields, including image processing [23], parameter tuning [24], machine learning (ML) [24], network optimization [25], signal compression [26,27], and numerous others due to their effectiveness and efficiency.

The MDPSO algorithm has been designed to handle problems where dimensionality is not fixed but varies within a defined range. In this method, dimensions or dimension indices can be identified within the interval $d_{\min}\leq d\leq d_{\max}$, allowing the algorithm to dynamically adapt to the challenges arising in various dimensions. Each particle in MDPSO undergoes two main processes: the first is the conventional, position-based optimization of PSO, involving standard velocity updates (Equation (1)) and position shifts (Equation (2)) within a dimension-indexed (*d*) search space. The second process is the dimensional PSO, which enables a particle to navigate between different dimensional spaces. During optimization, each particle stores its last position ($x^{d}$), velocity ($v^{d}$), and local best position (${\tilde{y}}^{d}$) in the given dimension so that if it returns to the same dimension later, it can continue the search from previously stored information. During the dimensional PSO process, a particle can switch to another dimension (with a different index), remembering its previous positional state in that dimension and continuing the optimization there. Meanwhile, the swarm tracks the best particle position vectors (${\hat{y}}^{d}$) in each dimension, each representing the best global position found so far in dimension *d*, which is used in the velocity update equation for that dimension:

(1) $$v_{k}^{d_{k}}\leftarrow c_{1}r_{1}\left({\tilde{y}}_{k}^{d_{k}}-x_{k}^{d_{k}}\right)+c_{2}r_{2}\left({\hat{y}}^{d_{k}}-x_{k}^{d_{k}}\right)+wv_{k}^{d_{k}}$$

Here, $v_{k}^{d_{k}}$ represents the velocity of the particle *k* in dimension $d_{k}$, controlling the particle’s movement within the search space. The coefficients $c_{1}$ and $c_{2}$ are the cognitive and social learning coefficients, respectively, which balance the influence of the particle’s own best experience and the collective knowledge of the swarm. Random numbers $r_{1}$ and $r_{2}$ are sampled uniformly from [0,1] to introduce stochastic behavior into the particle’s updates. The variable ${\tilde{y}}_{k}^{d_{k}}$ denotes the personal best position achieved by particle *k* in dimension $d_{k}$, while ${\hat{y}}^{d_{k}}$ represents the global best position found so far by the swarm in dimension $d_{k}$. *w* denotes the inertia weight of the velocity vector, which decreases linearly from 0.9 to 0.4 during iterations. This linear decrease helps facilitate the convergence of the algorithm [26,28].

The updated position of particle *k* in dimension $d_{k}$ is computed as

(2) $$x_{k}^{d_{k}}\leftarrow x_{k}^{d_{k}}+v_{k}^{d_{k}}$$

Similarly, the dimensional PSO process allows each particle to use its own best dimension (${\tilde{d}}_{k}$), where it achieved the best fitness value so far. The velocity for switching between dimensions is updated as

(3) $$vd_{k}\leftarrow c_{1}r_{1}\left({\tilde{d}}_{k}-d_{k}\right)+c_{2}r_{2}\left(\hat{d}-d_{k}\right)+w\;vd_{k}$$

The dimension index is then updated as

(4) $$d_{k}\leftarrow d_{k}+vd_{k}$$

Here, $vd_{k}$ represents the velocity for dimensional switching of particle *k*, enabling the particle to transition between different dimensions in the search space. The variable ${\tilde{d}}_{k}$ indicates the particle’s personal best dimension, where it has achieved the best fitness value so far. Lastly, $\hat{d}$ denotes the best global dimension found by the swarm, representing the dimension where the overall best solution has been identified during the optimization process. Finally, the swarm tracks the best global dimension ($\hat{d}$) among all the best local dimensions. In the last iteration, the best particle ($x_{gBest^{\hat{d}}}^{\hat{d}}$) found in the dimension considered optimal ($\hat{d}$) represents the optimal solution.

In the presented pseudocode (Algorithm 1), we use the following naming conventions:

- *N* denotes the number of iterations performed during the search process;
- gBest stores the index of the particle that achieves the best result;
- *S* is the total number of particles in the swarm, and *k* is the index of the current particle;
- ρ(·,·) is a vector-valued real loss function that optimizes the solution to the given problem while considering the dimensionality of the search space (see, e.g., Algorithm 2).

### 3.2. The Proposed Algorithm for 2D and 3D Image Clustering

Our segmentation approach is based on multidimensional particle swarm optimization (MDPSO) and draws inspiration from K-means [18] and mean shift-based methods [19]. Each particle in the swarm encodes a candidate segmentation of either a 2D or 3D MRI image with four MRI modalities (FLAIR, T1C, T1, and T2). We segment the image into *d* clusters, each represented by a centroid in a joint spatial-intensity feature space. Specifically, for a spatial dimensionality *S*, where S=2 for 2D images (slices) and S=3 for 3D volumes, each centroid consists of *S* spatial coordinates and the intensity values of the M=4 modalities. Thus, the dimensionality of the search space is (S+M)×d, being 6d for the 2D case and 7d for the 3D case.

At the beginning of the segmentation process, the *d* centroids are initialized uniformly and independently within the normalized spatial domain. The intensity attributes of each centroid are initialized by sampling the image intensity values at random positions. During segmentation, each image element—whether a 2D pixel or a 3D voxel (for S=3)—at spatial coordinate $x_{p}=\left(x_{p,1},\;x_{p,2},\;{\left(x_{p,3}\right)}_{S=3}\right)$ is represented by the feature vector

$$p=\left[x_{p,1},\;x_{p,2},\;{\left(x_{p,3}\right)}_{S=3},\;I_{\mathrm{flair}}\left(x_{p}\right),\;I_{t1c}\left(x_{p}\right),\;I_{t1}\left(x_{p}\right),\;I_{t2}\left(x_{p}\right)\right].$$

It is then assigned to its nearest centroid

$$x_{c}^{d}=\left[x_{c,1},x_{c,2},{\left(x_{c,3}\right)}_{S=3},I_{\mathrm{flair}}\left(x_{c}\right),I_{t1c}\left(x_{c}\right),I_{t1}\left(x_{c}\right),I_{t2}\left(x_{c}\right)\right],$$

based on a weighted, transformed distance metric. The distance measure is derived using a transformation function f(·), which is applied to the absolute differences of attributes as follows:

(5) $$f:\left[0,\infty \right)\to \left[0,\infty \right),\;f\left(x\right)=e^{x^{2}}-1.$$

Each attribute difference $|p_{m}-x_{c,m}^{d}|$ is scaled by a factor $\alpha_{m}$ and weighted by a factor $w_{m}$ after applying the transformation f(·). Consequently, the distance from an element p to centroid $x_{c}^{d}$ is calculated as

(6) $$\delta_{c}\left(p\right)=\sum_{m=1}^{S+M}w_{m}\;f\left(|p_{m}-x_{c,m}^{d}|\cdot \alpha_{m}\right).$$

After assigning each image element p to the closest centroid c(p), we compute the cluster-wise error sums. For cluster *i*, we define

$$P_{i}=\left\{p|c\left(p\right)=i\right\},\;A_{i}=\left|P_{i}\right|,\;e_{i}=\sum_{p\in P_{i}}\delta_{i}\left(p\right),$$

where $A_{i}$ is the number of image elements in cluster *i*, and $e_{i}$ is the cumulative transformed error within that cluster. Using these values, the global fitness measure, which the algorithm seeks to minimize, is defined as

(7) $$fitness=\frac{\sqrt{d}}{A}\sum_{i=1}^{d}\frac{e_{i}^{2}}{\sqrt{A_{i}}},$$

where *A* is the total number of pixels (for 2D) or voxels (for 3D) in the image. Adapted from [29], this formulation incorporates a $\sqrt{d}$ factor and divides by $\sqrt{A_{i}}$, ensuring neither excessively fine nor overly coarse segmentations are trivially favored.

This global fitness measure integrates into the MDPSO framework, where each particle’s position in the (S+M)d-dimensional space defines a complete set of centroids. Over iterations, centroid positions are refined according to the inertia, cognitive, and social components of MDPSO, with velocity clamping ensuring stable exploration of the solution space. Dimensionality changes (*d*) are regulated through controlled particle entry into new dimensions, following the approach suggested in [24]. Through the transformations in (5) and (6), and the normalization in (7), the method adaptively segments the MRI images. Lower fitness values indicate improved segmentation quality, effectively balancing the number of clusters and error minimization.

By maintaining this adaptive balance, the approach prevents excessively coarse or overly fragmented segmentations, facilitating an optimal clustering solution for both 2D slices and 3D volumetric MRI data.

### 3.3. Complexity Analysis

The overall computational cost of the MDPSO algorithm (Algorithm 1) can be analyzed by considering the following factors. The algorithm performs *N* iterations of the MDPSO procedure. At each iteration, a swarm of *S* particles is processed. Each particle explores multiple clusterings parameterized by $d\in \{d_{\min},\cdots ,d_{\max}\}$, resulting in $K\;=\;d_{\max}\;-\;d_{\min}\;+\;1$ different configurations per particle. For each configuration, the fitness function $\rho (x^{d},d)$ (Algorithm 2) is evaluated.

The computational complexity of a single evaluation of the fitness function ρ depends primarily on the number of image elements *A* (i.e., pixels in 2D images or voxels in 3D volumes) and the number of clusters, which is determined by *d*. For each image element, the algorithm computes its distance to each centroid and assigns it to the nearest one, resulting in a complexity of

O(A·d)

per fitness evaluation.

The total number of fitness evaluations includes those performed during the initialization phase and throughout the *N* optimization iterations. During initialization, each of the *S* particles is evaluated for all clusterings from $d_{\min}$ to $d_{\max}$. This accounts for

O(S·K)

fitness evaluations. During the optimization phase, each of the *S* particles is updated and re-evaluated across iterations, contributing an additional

O(N·S)

fitness evaluations.

Combining these, the total number of evaluations is

$$O\left(S\cdot (K+N)\right).$$

Since each fitness evaluation incurs a cost of O(A·d), the overall computational complexity becomes

$$O\left(S\cdot (K+N)\cdot A\cdot d\right),$$

where *d* is considered as a representative or average cluster count in the range $\left[d_{\min},d_{\max}\right]$. However, practically, the algorithm does not necessarily evaluate all clusterings at every iteration beyond initialization, potentially reducing effective computational demands.

From a practical implementation perspective, the algorithm is inherently parallelizable. Particle positions and fitness evaluations for multiple particles and multiple image elements can be computed concurrently, efficiently leveraging multi-core CPUs, GPUs, or other parallel processing architectures.

### 3.4. Metrics

To quantitatively evaluate clustering quality in our segmentation approach based on clustering, we adopted a modified version of the Achievable Segmentation Accuracy (ASA) metric. ASA, originally proposed as an upper-bound measure of segmentation quality, assigns each predicted segment (cluster) to the ground truth region with which it shares the largest overlap [30]. It has been widely employed to assess the quality of unsupervised segmentation and superpixel decomposition tasks [31].

The original ASA metric is defined as follows:

(8) $$\mathrm{ASA}\left(c\right)=\frac{\sum_{k}\max_{i}\left|c_{k}\cap g_{i}\right|}{\sum_{i}\left|g_{i}\right|},$$

where $c_{k}$ denotes the *k*-th predicted segment (cluster) in segmentation *c*, and $g_{i}$ represents the *i*-th ground truth region.

Given the significant class imbalance typically observed in lesion segmentation tasks, we propose a Dice-like variant of ASA, denoted as ${\mathrm{ASA}}_{\mathrm{Dice}}^{\left(L\right)}\left(c\right)$, to explicitly penalize both false negatives and false positives. In this formulation, each predicted cluster is labeled as either lesion or background according to the majority label of its constituent voxels. The modified metric is defined as

(9) $${\mathrm{ASA}}_{\mathrm{Dice}}^{\left(L\right)}\left(c\right)=\frac{2\cdot \sum_{k\in L}\max_{i\in G_{L}}\left|c_{k}\cap g_{i}\right|}{\sum_{k\in L}|c_{k}|+\sum_{i\in G_{L}}\left|g_{i}\right|},$$

where the following applies:

- L is the set of predicted clusters labeled as lesions;
- $c_{k}$ denotes a predicted cluster classified as a lesion (k∈L);
- $G_{L}$ is the set of ground truth lesion regions;
- $g_{i}$ represents the *i*-th lesion region in the ground truth ($i\in G_{L}$).

The proposed ${\mathrm{ASA}}_{\mathrm{Dice}}^{\left(L\right)}\left(c\right)$ retains the core principle of ASA—emphasizing maximal overlap between predicted segments and ground truth regions—while incorporating Dice-like normalization to more effectively address class imbalance. By specifically considering overlaps related to lesion regions, this metric offers a more informative and balanced assessment of clustering-based segmentation performance in unsupervised lesion segmentation scenarios.

For supervised evaluation, we additionally compute the following standard metrics:

(10)    Dice=2TP2TP+FP+FN;(11)  Precision=TPTP+FP;(12)   Sensitivity=TPTP+FN;(13)  Accuracy=TP+TNTP+TN+FP+FN;(14)   Specificity=TNTN+FP;(15)Hausdorff95=maxP95{minb∈B∥a−b∥:a∈A},P95{mina∈A∥b−a∥:b∈B},

where TP, TN, FP, and FN are true positives, true negatives, false positives, and false negatives, respectively; *A* and *B* represent the boundary points of the predicted and ground truth segmentations; and $P_{95}$ denotes the 95th percentile of the respective distance distributions.

## 4. Experiments and Discussion

### 4.1. Dataset

The MRI data originate from the RSNA-ASNR-MICCAI BraTS challenges, providing diverse multiparametric MRI scans of gliomas from various institutions [32,33,34] (for data sources, see Section 5). Each case includes native and post-contrast T1-weighted, T2-weighted, and T2-FLAIR volumes, acquired using different clinical protocols and scanner types. This inherent heterogeneity supports the development of data-independent models robust to inter-institutional and scanner variability. The BraTS dataset was selected not only for its size and diversity but also for the high quality of its annotations, which have been iteratively refined to reduce interobserver variability. This refinement increases annotation reliability, which is essential for developing generalizable segmentation models. A key goal of this study is to evaluate how variability in acquisition protocols and institutional sources affects segmentation performance.

For 2D segmentation, we extracted 335 axial slices from the BraTS 2021 release. Of these, 200 slices were used for training, 100 for testing, and 35 for validation. All slices were taken from the 77th (middle) axial plane to ensure a consistent anatomical perspective and reduce slice-level variability. Histogram equalization was applied independently to each volume via a linear transformation [35]. Zero-intensity pixels were labeled as background; non-zero intensities were normalized such that the 25th percentile mapped to 0.4 and the 75th percentile to 0.6, then clipped to the [0,1] range.

For 3D segmentation, we used the BraTS 2019 dataset. The same 200:35:100 train–validation–test split was applied across 335 volumes. To make volumetric processing tractable, we employed superpixel-based decomposition using the SLIC algorithm with hyperparameters from Amendola et al. [31], yielding k=5000 superpixels per volume and a compactness parameter of 5.0. This configuration achieved an ${\mathrm{ASA}}_{\mathrm{Dice}}^{\left(L\right)}$ score of 93.87 % (see Section 3.4), demonstrating that superpixel decomposition via SLIC effectively reduces data complexity while preserving segmentation quality.

### 4.2. Parameter Settings

#### 4.2.1. Clustering Parameters

In our experiments, several algorithmic parameters were manually configured to ensure a balance between computational efficiency and segmentation performance for both 2D and 3D data. For 2D segmentation, the clustering algorithm was executed with a swarm size of S=64. For 3D segmentation, where the spatial complexity and search space are significantly greater, the swarm size was increased to S=128. In both settings, the number of iterations was fixed at N=15, which was empirically found to provide reliable convergence without incurring unnecessary computational overhead.

The number of centroids was controlled by a discrete index *d*, varied within task-specific bounds. In the 2D case, this index was constrained to the interval d∈{5,⋯,10}, while for 3D segmentation, it was extended to d∈{15,⋯,30} to accommodate the increased representational requirements of volumetric data. To enhance clustering granularity without modifying the underlying framework, the number of centroids was defined as 2d for both configurations.

Regarding swarm population size, we adhered to the guidelines established in [36], which suggest that sizes in the range of 20 to 50 generally yield effective performance. Empirical validation confirmed that increasing the swarm size beyond 50 produced no measurable improvement in segmentation accuracy.

**Figure 2:**
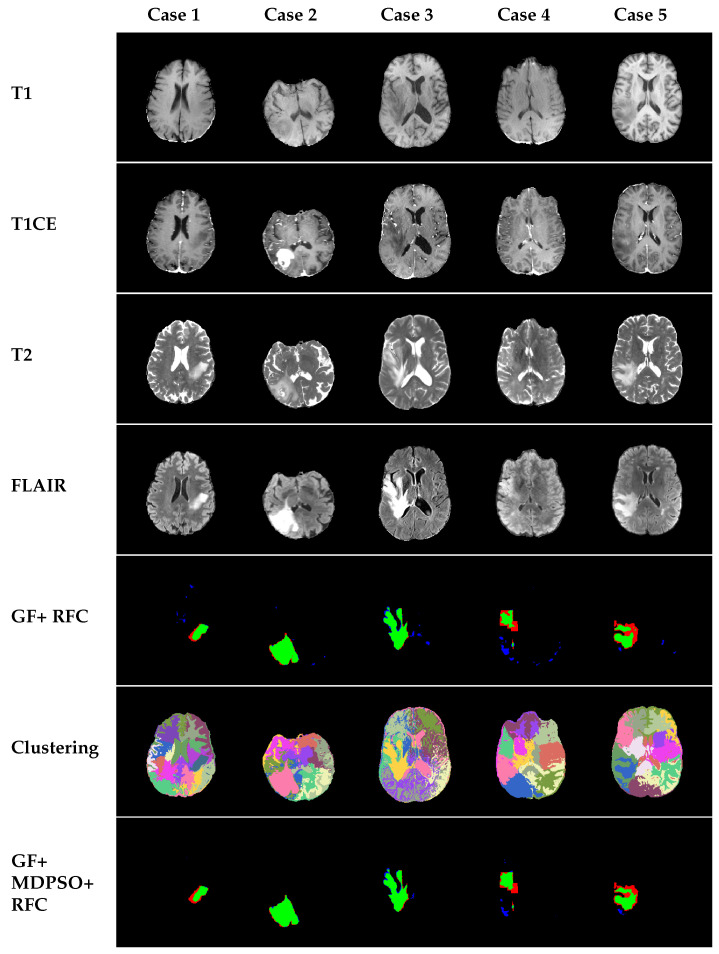
Examples from the dataset showcasing different MRI modalities and 2D segmentation evaluations performed on BraTS2021 dataset slices. The rows indicate the image modality or evaluation type, while the columns correspond to individual cases. Specifically, the rows represent T1, T1CE, T2, FLAIR, and RFC results using only GF features, clustering results via MDPSO, and the final segmentation results combining clustering and GF features (our proposed method). True positive (TP) pixels are shown in green, false positive (FP) pixels are shown in blue, and false negative (FN) pixels are shown in red.

**Figure 3:**
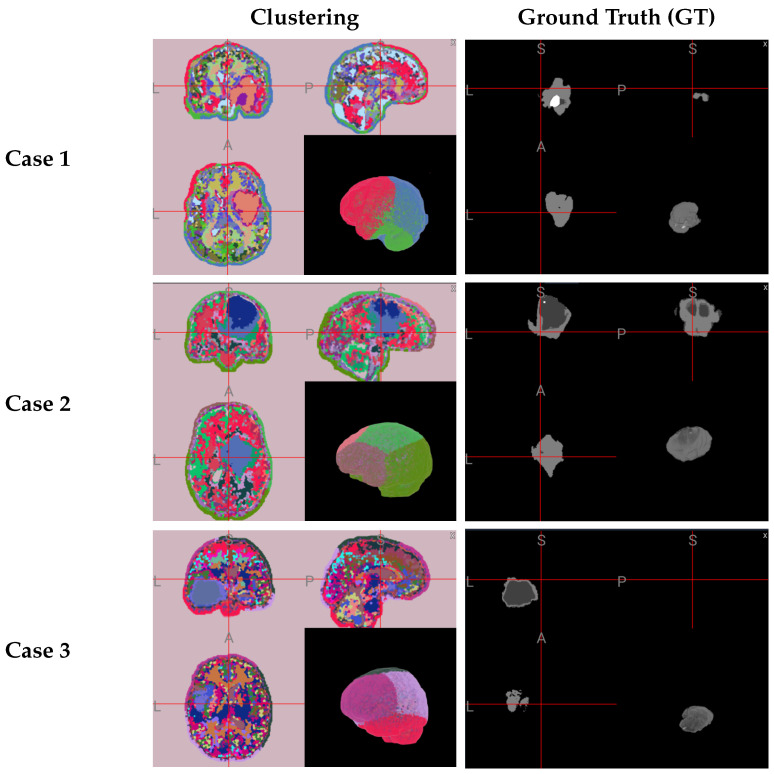
Three-dimensional visualization of clustering results versus ground truth for representative subjects from the BraTS 2019 dataset. Each panel includes three orthogonal slices arranged as follows: coronal view (top-left), sagittal view (top-right), and axial view (bottom-left), along with a 3D rendering in the bottom right. The clustering column uses a random color scale to enhance contrast between different regions. In the ground truth images, white indicates enhancing tumor (ET), light gray represents edema (ED), and dark gray corresponds to non-enhancing tumor core (NCR/NET).

Automated calibration of the model parameters $\alpha_{m}$ and $w_{m}$ from Equation (6) was performed using the Bayesian Tree-structured Parzen Estimator (TPE). The optimization was conducted on a set of 40 representative 2D slices selected from the training dataset. The search domain was defined as ${\left[0,5\right]}^{6}$, covering six tunable parameters, with the objective of maximizing the ${ASA}_{\mathrm{Dice}}^{\left(L\right)}$ metric defined in Equation (9). This metric quantifies the spatial overlap between predicted clusters and annotated tumor regions, promoting solutions that delineate tumor substructures with high fidelity. During optimization, the Euclidean distance weight $\alpha_{\mathrm{dist}}$ was allowed to vary, while the intensity-based weight was fixed at unity. The spatial weights $w_{m}$ were constrained to remain isotropic across spatial dimensions. The FLAIR modality was consistently assigned the highest feature weight, underscoring its effectiveness in visualizing tumor regions [3]. The final parameter configurations resulted in accurate and anatomically coherent segmentation outputs in both 2D and 3D tasks, as illustrated in Figure 2 and Figure 3.

#### 4.2.2. Random-Forest Feature Extraction and Training

A randomized, overcomplete 3D GF bank was initially constructed by continuously sampling filter parameters across scales, frequencies, and orientations. To mitigate redundancy, filters were vectorized and clustered via *k*-means (k=75), from which two representative filters per cluster were selected based on either maximal discriminative power (measured by absolute mean response difference between tumor and non-tumor voxels) or maximal response variance across voxels from the training dataset. Each filter was subsequently applied exclusively to the MRI modality yielding the highest informational content. This resulted in a set of 150 modality-specific GFs, with most associated with the FLAIR modality due to its superior contrast properties for tumor discrimination.

Extracted filter responses were then combined with voxel-level descriptors, including raw MRI intensities, Gaussian local standard deviations, median-filtered intensities, and a comprehensive set of cluster-level descriptors. Cluster-level descriptors integrated geometric, spatial, and intensity-based characteristics, comprising 24 modality-independent features (e.g., fractal dimension, convexity, centroid coordinates, spatial moments, cluster density, and eccentricity) and 16 modality-dependent features capturing the intensity and textural attributes (mean intensity, standard deviation, contrast, homogeneity) across each MRI modality. Consequently, the final feature dimensionality totaled 218 for 3D and 212 for 2D contexts. For both the 2D and 3D segmentation models, 10,000 voxels per volume or slice were randomly sampled, providing two million training samples in total. Independent random forest classifiers consisting of 500 trees were trained for each dimensionality, enabling voxel-wise tumor segmentation predictions. The remaining hyperparameters were selected using the TPE optimization method, with trial models evaluated on the validation dataset. Source code and additional implementation details are available at https://github.com/bogazsombor/MDPSO_for_MRI_segmentation.

### 4.3. Results

#### 4.3.1. Qualitative Results

In Figure 2, we present some evaluation results of the model when pixel classification is performed solely based on GF features (GF + RFC). In the last row, we display the performance of our proposed segmentation algorithm, which integrates clustering and GF features (GF + MDPSO + RFC). Comparing the two methods, we observe that our approach, which introduces contextual information through cluster-based features, effectively reduces the number of scattered false-positive pixels in the segmented images.

Further qualitative evaluation through 3D visualization (Figure 3) highlights the robustness and effectiveness of the MDPSO clustering algorithm, also in 3D. Despite being largely unsupervised—with only minimal hyperparameter tuning applied across a limited number of cases—the algorithm consistently differentiates not only between healthy and lesional tissues but also among distinct tumor subregions. The combination of spatial coherence and color encoding generates a feature space conducive to separating anatomically and pathologically meaningful regions. Clusters associated with tumors exhibit distinct patterns of homogeneity and sparsity compared to those representing healthy tissue, and different subregions of the tumor (e.g., enhancing core, edema, and necrotic core) are often separated into individual clusters. While some variability exists at cluster boundaries, these can be further refined through local textural analysis. Overall, the integration of MDPSO-based clustering with Gabor feature-driven RFC results in coherent and interpretable segmentations, underscoring the method’s potential for robust and clinically relevant brain tumor delineation.

#### 4.3.2. Quantitative Results

Integrating contextual information through MDPSO-derived cluster features significantly enhanced segmentation performance in both 2D and 3D analyses (Table 1).

All statistical tests were evaluated against a pre-specified significance level of α=0.05. In 2D segmentation, median Dice improved notably from 85.3% (IQR: 22.0) using GF+RFC alone to 87.6% (IQR: 14.9) with additional MDPSO features, reflecting not only improved performance but also greater consistency. These improvements were statistically significant according to the Wilcoxon signed-rank test (W=930, p<0.001<0.05), where *W* is the smaller of the sums of the positive- and negative-difference ranks. Similarly, in 3D segmentation, median Dice rose from 84.8% (IQR: 19.3) to 86.7% (IQR: 15.2); this increase was also significant (W=820, p<0.001<0.05).

Beyond numerical improvements, clinically meaningful classification accuracy was also enhanced. The number of successful segmentations (Dice ≥ 85%) increased significantly in both 2D (p=0.029<0.05) and 3D (p=0.043<0.05) analyses, as determined by McNemar’s test. Thus, incorporating MDPSO-derived cluster features not only improves overall segmentation accuracy but also significantly reduces critical segmentation errors, reinforcing their practical value for clinical applications.

### 4.4. Comparison with Other Approaches

In developing our method pipeline, we built upon the approach described in [9], and the results (RF + GF) are very similar to those reported therein. However, our method has been extended with several additional valuable cluster features. Specifically, our MDPSO-based clustering approach has been refined to group image pixels that are similar in color and are physically near to each other. This clustering process enables the extraction of detailed information regarding the density of similar pixels within a region, the spatial extent they cover, and the shape and edge characteristics of the resulting clusters. Such an approach mimics the human visual perception process and is particularly advantageous in the field of medical imaging, where explainable decision-making is critical. Our experimental results indicate that these properties are, indeed, capable of enhancing segmentation accuracy metrics.

In contrast, many CNN models suffer from a lack of interpretability and are typically considered blackbox solutions. Although these models often demonstrate high performance, their decisions are generally verified only through extensive testing or post-hoc analysis. As shown in Table 2, our proposed method achieves whole-tumor segmentation performance that is comparable to state-of-the-art machine learning and deep learning models. While our results might be slightly lower than those of the most data-intensive deep learning methods (e.g., [3,10]), our approach requires significantly fewer parameters and less training data.

Furthermore, recent transfer learning methods have shown promise in reusing knowledge from different domains, thereby reducing the amount of data required for training [11]. Despite this advantage, their segmentation performance remains inferior to that of conventional methods that utilize large amounts of data, and our approach surpasses these methods as well.

We also explored other metaheuristic clustering algorithms, which sometimes achieve high accuracy; however, they typically require human intervention after clustering to decide which clusters truly represent tumor regions [37,38]. To the best of our knowledge, our proposed method is the first to use the result of a metaheuristic clustering algorithm to enable fully automated decision-making by incorporating a PSO algorithm. A major benefit of our approach is that it does not require the number of clusters to be specified in advance; instead, the algorithm automatically determines the optimal number of clusters.

## 5. Conclusions and Future Directions

In conclusion, our application of the MDPSO algorithm for image segmentation has demonstrated that robust and accurate results can be achieved with minimal reliance on annotated data. Unlike conventional thresholding techniques, our approach is capable of detecting objects and autonomously determining the optimal number of segments. This highlights the potential of semi-supervised and unsupervised methods in reducing the dependence on time-consuming expert annotations.

Future work will initially focus on exploring additional supervised learning approaches, particularly those that can effectively leverage information derived from unsupervised algorithms and operate with a reduced number of annotated samples. This aims to further enhance segmentation accuracy and computational efficiency. Subsequent efforts will concentrate on minimizing data dependency by progressively transitioning toward fully unsupervised frameworks, which offer improved robustness against variations in MRI acquisition protocols. Ultimately, our goal is to develop trustworthy, interpretable, and generalizable unsupervised segmentation methods that can be integrated into clinical practice and extended to other medical imaging domains where annotated data is scarce, thereby promoting broader and more efficient adoption of automated segmentation workflows.

## Figures and Tables

**Table 1:** Performance metrics (mean, median, and inter-quartile range) computed over 100 test images for RFC-based segmentations. The first and third rows show results from RFC segmentations based solely on GF features (in 2D and 3D, respectively), while the second and fourth rows show results when MDPSO-derived cluster features are added to the RFC input (for 2D and 3D, respectively). Bold indicates the better result in each pair of experiments, demonstrating statistical improvements—higher mean and median and lower IQR—in the extended version with MDPSO features.

Method	Statistic	Dice (%)	Precision (%)	Sensitivity (%)	Accuracy (%)	Specificity (%)	Hausdorff95 (mm)
GF+ RFC (2-D)	Mean	81.92	88.89	77.20	95.02	99.20	19.34
	Median	85.32	90.50	81.11	97.84	99.32	7.00
	IQR	22.01	12.37	26.88	2.39	0.92	11.01
GF+ MDPSO+ RFC (2-D)	Mean	**84.94**	**91.87**	**81.22**	**97.72**	**99.31**	**10.41**
	Median	**87.64**	**93.81**	**85.37**	**98.16**	**99.41**	**6.21**
	IQR	**14.88**	**8.17**	**20.23**	**1.69**	**0.81**	**7.87**
GF+ RFC (3-D)	Mean	82.55	87.62	78.43	96.21	98.94	23.68
	Median	84.81	89.05	80.02	97.02	99.08	9.55
	IQR	19.34	11.48	22.77	2.12	1.12	13.73
GF+ MDPSO+ RFC (3-D)	Mean	**84.37**	**90.42**	**80.51**	**97.11**	**99.12**	**15.28**
	Median	**86.72**	**91.63**	**83.77**	**97.88**	**99.27**	**8.33**
	IQR	**15.21**	**9.84**	**19.59**	**1.88**	**0.97**	**9.44**

**Table 2:** Overview of various recent brain tumor segmentation methods (2021–2025). The Dataset column provides data source details, including dataset size, train–test split, and imaging dimensionality. The Dice Score column reports segmentation performance as mean Dice scores; some studies further report scores for specific regions (e.g., Enhancing Tumor (ET), Tumor Core (TC), Whole Tumor (WT), or by tumor types such as high-grade glioma (HGG) or low-grade glioma (LGG)). The Train & Inference Time column lists training duration and per-volume or per-image inference time (N/R indicates not reported). The Hardware column describes the computing platform used, listing CPU and GPU explicitly. The Year column indicates the publication year.

Method (Type)	Dataset (Train & Test Size, Dimensionality)	Dice Score (%)	Train & Inference Time	Hardware	Year
Binary Decision Trees Ensemble [9]	BraTS2019 (76 LGG, 259 HGG, 5:1 split, 3D MRI)	LGG: 84.79 HGG: 85.16	Train: N/R Test: 58 s/vol.	CPU: Intel Core i7, 16 GB RAM	2021
nnU-Net (3D U-Net with modifications) [10]	BraTS2021 (1251/219, 3D MRI)	ET: 84.51 TC: 87.81 WT: 92.75	N/R	GPU: NVIDIA RTX 3090(24 GB)	2021
Cascade CNN + Distance-Wise Attention [12]	BRATS2018 (75 LGG, 210 HGG, 9:1 split, 3D MRI)	WT: 92.03 ET: 91.13 TC: 87.26	Train: 13 h, Test: 7 s/vol.	CPU: Intel Core i7, 32 GB RAM, GPU: NVIDIA GeForce GTX 1080Ti	2021
nnU-Net–based Medulloblastoma Segmentation [11]	Local data from 3 inst. (78 cases, leave-one-institution-out split strategy, 2D MRI)	84.33	N/R	N/R	2024
EHO-EnFCM [37]	BraTS (20 images, 3D MRI)	80.07	Train: N/R Test: 26.57 s/img.	CPU: Intel Core i7, 8 GB RAM	2025
MDPSO + RFC for 2D images (Ours)	BraTS2021 (235/100, 2D MRI)	84.94	Train: 1.5 h, Test: 22 s/img.	GPU: NVIDIA GeForce RTX 3060 (12 GB)	2025
MDPSO + RFC for 3D data (Ours)	BraTS2019 (235/100, 3D MRI)	84.37	Train: 3 h, Test: 37 s/img.	GPU: NVIDIA GeForce RTX 3060 (12 GB)	2025

## Data Availability

The datasets used in this study are publicly available from the RSNA-ASNR-MICCAI Brain Tumor Segmentation (BraTS) challenges [32,33,34]. BraTS 2021 dataset: https://www.kaggle.com/datasets/dschettler8845/brats-2021-task1 (accessed on 2 October 2023). BraTS 2019 dataset: https://www.kaggle.com/datasets/aryashah2k/brain-tumor-segmentation-brats-2019 (accessed on 2 October 2023).
